# Use and Interpretation of a Rapid Respiratory Syncytial Virus Antigen Detection Test Among Infants Hospitalized in a Neonatal Intensive Care Unit — Wisconsin, March 2015

**DOI:** 10.15585/mmwr.mm6431a6

**Published:** 2015-08-14

**Authors:** Lina I. Elbadawi, Thomas Haupt, Erik Reisdorf, Tonya Danz, Jeffrey P. Davis

**Affiliations:** 1 Epidemic Intelligence Service, CDC; 2 Division of Public Health, Wisconsin Department of Health Services; 3 Wisconsin State Laboratory of Hygiene

On March 25, 2015, the Wisconsin Division of Public Health was notified of a possible respiratory syncytial virus (RSV) infection outbreak among infants hospitalized in a neonatal intensive care unit (NICU). On March 23, the index patient (neonate A), aged 3 days, had feeding intolerance and apnea. A nasopharyngeal swab specimen collected from neonate A was tested using a single-manufacturer rapid RSV antigen detection test (RRADT) at the hospital laboratory; the result was positive. The following day, because of concern about the possibility of more widespread RSV infection, RRADT was used to test nasopharyngeal swab specimens from neonate B, aged 1 month, who had resided in a different hospital room in the NICU and had developed an increased oxygen requirement, apnea, and poor feeding that day, as well as from two asymptomatic neonates who were hospitalized in the same room with neonate A; all three were positive. Later that day, nasopharyngeal swab specimens from the remaining 16 asymptomatic NICU patients were tested using the same RRADT; seven tests were positive, making a total of 11 positives. All 20 RRADTs were performed at the hospital laboratory.

On March 25, the same 20 nasopharyngeal specimens were sent to the Wisconsin State Laboratory of Hygiene for confirmatory testing using a multiplex respiratory virus real-time polymerase chain reaction (PCR) panel (eSensor, GenMark Diagnostics, Inc.) that targets 18 viruses, including RSV subgroups A and B. Sixteen nasopharyngeal specimens were negative for all 18 virus targets; three were positive for RSV-A, including the specimens from neonates A and B and from one asymptomatic neonate whose RRADT result was positive. A nasopharyngeal swab specimen from one other asymptomatic neonate with a positive RRADT tested positive for human coronavirus 229E by PCR. All nasopharyngeal specimen PCR results were confirmed at CDC. Therefore, among 17 specimens that were RSV-negative by PCR, eight were positive by RRADT, for a false-positivity rate of 47%.

The sensitivity (percentage of persons with the disease who have a positive test) and specificity (percentage of persons without the disease who have a negative test) of RRADTs for detecting RSV are characteristics of the test. However, test result interpretation depends on the positive predictive value (PPV) (i.e., the proportion of test-positive patients who have RSV infection), which is influenced by RSV infection prevalence. Studies among infants and young children with symptoms consistent with respiratory illness during peak RSV season (late January through March) demonstrated a sensitivity, specificity, and PPV for RRADT of 80%–85%, 96%–100%, and 85%–100%, respectively (1–3). However, the reported PPV of a test might not be applicable if the patient being tested is dissimilar to the population evaluated to determine the PPV; in this case, the PPV of a test used on symptomatic infants might not necessarily apply to asymptomatic infants, even if both are tested during peak RSV season.

Other possible contributors to the high rate of false positives include contaminated viral transport media or applied topical preparations, such as emollients to the neonates’ nares. Aliquots from all infant nasopharyngeal specimens were provided to the RRADT manufacturer without personal identifying information for validation and verification; testing of these specimens was conducted by the manufacturer, and the hospital laboratory RRADT results were replicated.

At the conclusion of the investigation, Wisconsin Division of Public Health recommended to the facility that the RRADT be used only for testing symptomatic neonates in accordance with manufacturer guidelines. In addition, the division recommended that any positive RRADT results be confirmed by real-time PCR that would detect RSV A and B. Diagnostic tests indicated for use in patients with a characteristic clinical illness might produce misleading results if used for another purpose, such as for screening of asymptomatic patients.
